# “The thing I’m missing the most is just being around other queer people”: critical analysis of the impacts of the COVID-19 pandemic on mental health of two-spirit, gay, bisexual, and queer men’s communities in Manitoba, Canada

**DOI:** 10.1186/s12889-023-16205-6

**Published:** 2023-07-04

**Authors:** Rusty Souleymanov, Samantha Moore, Jared Star

**Affiliations:** 1 Department of Social Work, Faculty of Social Work, University of Manitoba, 173 Dafoe Road West, Tier Building, Office 500 C, Winnipeg, MB R3T 2N2 Canada; 2 Department of Community Health Sciences, University of Manitoba, Winnipeg, MB Canada

**Keywords:** 2SGBQ + health, COVID-19, Mental health, Social isolation, Place, Biosexual citizenship, Queer identity

## Abstract

**Background:**

The purpose of this community-based study was to create and advance knowledge on the social impacts of COVID-19 on mental health of Two-Spirit, gay, bisexual, and queer (2SGBQ+) cisgender and transgender men in Manitoba, Canada.

**Methods:**

Participants (n = 20) from 2SGBQ + men’s communities were recruited across Manitoba using printed flyers and social media. Individual interviews explored questions relating to the impacts of the COVID-19 pandemic on mental health, social isolation, and service access. Data were critically examined using thematic analysis and the social theory of biopolitics.

**Results:**

Key themes focused on COVID-19 pandemic’s negative impacts on 2SGBQ + men’s mental health, loss of safe queer public spaces, and exacerbated inequities. During the COVID-19 pandemic in Manitoba, 2SGBQ + men experienced a profound loss of social connections, community spaces, and social networks which are specific to their socio-sexual identities, thereby intensifying pre-existing mental health disparities. These findings show how COVID-19 restrictions have come to reinforce the value of close personal communities, families of choice, and social networks among 2SGBQ + men in Manitoba, Canada.

**Conclusions:**

This study supports the line of research on minority stress, biosociality, and place by highlighting some potential links between 2SGBQ + men’s mental health and their social and physical environments. This research points to important role of safe community spaces, events, and community organizations that support 2SGBQ + men’s mental health.

## Introduction

The COVID-19 pandemic has caused innumerable shifts in the everyday lives of Canadians since the first governmental restriction mandates were imposed in the early months of 2020. Gathering restrictions, social distancing protocols, and other lockdown measures have negatively impacted the health of marginalized populations, such as Two-Spirit, gay, bisexual, and queer (2SGBQ+) cisgender and transgender men [1, 2]. These restrictions also interrupted the ability of health and social service providers to meet the needs of vulnerable clientele and created barriers in accessing informal support networks that often provided a sense of community and identity for 2SGBQ + men [1, 2]. 2SGBQ + men are a population who may be uniquely impacted by COVID-19 restrictions, which therefore warrants an investigation. In Canada, 2SGBQ + men are already at a disproportionately high risk for a variety of negative mental health outcomes, such as depression, anxiety, loneliness, and social isolation [3, 4]. In one Manitoba study of 2SGBQ + men conducted in 2019, 52% of participants (*n* = 410) reported that they needed help with problems like anxiety, depression, and suicidal thoughts [5]. This implies that mental health challenges among 2SGBQ + men were already prevalent and deserving of attention even prior to the COVID-19 pandemic. Furthermore, social isolation, limited access to friends and partners, and sheltering in hostile (homophobic and transphobic) home environments during COVID-19 is associated with poorer mental health outcomes among 2SGBQ + men [6–8]. Research among 2SGBQ + men also points to the adverse impacts of COVID-19 on mental health, substance use, quality of life, social isolation, and access to health services [9]. Yet, qualitative research on the impacts of COVID-19 restrictions on 2SGQ + men’s mental health is currently sparse. Qualitative research enables the exploration of the social, cultural, and structural factors that shape the mental health experiences of 2SGBQ + men during the pandemic. This study examines the experiences of 2SGBQ + men within Manitoba during the COVID-19 pandemic, with particular attention paid to the relationships among community belonging, mental health, and minority-based stress for 2SGBQ + men in Manitoba during the COVID-19 pandemic. We concentrate not only on experiences of social isolation due to quarantine, social distancing measures, and gathering restrictions, but also on the loss of queer spaces and the myriad of forms that such spaces can take, whether formal or informal. This loss of community connection is therefore explored within multiple realms; the social, physical, political, and symbolic. Additionally, we examine the ways in which 2SGBQ + men within Manitoba utilize both personal and social resourcefulness in the face of community loss when maneuvering everyday life during the pandemic.

### Queer spaces, community belonging and mental health among 2SGBQ + men

Queer spaces and queer connectedness have played a central role in the lives of 2SGBQ + men within Manitoba. Through the 1930’s to the 1960’s, the city of Winnipeg acted as the “queer capital” for not only residents of Manitoba, but also for those residing in Northwestern Ontario and parts of Saskatchewan [10]. The prairie city offered several physical spaces that provided safety and seclusion, where “many of the spaces [within] this subaltern queer world – the baths, cruising sites, the YMCA, or drag venues – encouraged and respected anonymity” [10]. From the 1960’s into the 1980’s, Winnipeg continued to act as a space for queer expression and community. Activism and political dissent came to shape a large part of 2SGBQ + identity in the city during the later decades of the 20th century and such engagement continues to the present day [11].

For 2SGBQ + men within the province of Manitoba, queer spaces that provide safety and acceptance, including health care facilities and informal meeting/leisure spaces, are of importance in minimizing experiences of discrimination while simultaneously uplifting experiences of autonomy, community, and activism [1, 12, 13]. Manitoba currently lacks many queer-friendly spaces more common in larger metropolitan areas.

Moreover, 2SGBQ + men may often turn to more informal support networks of friends and community members when normative familial structures fail to provide a sense of belonging, or when home life proves to be unsafe [8, 14]. Research that examines health-related aspects of queer socializing also includes conflicting findings; some scholars claim that residence in neighborhoods with high proportions of same-sex households is protective regarding health, while other research suggests that while queer institutions, such as bars or dance clubs, serve as safe venues for expression and socialization, they also facilitate health risk behaviors, such as substance use or condomless anal intercourse [15]. Research also suggests that a sense of belonging for queer individuals is generally regarded as important for mental health [16]. McLaren and colleagues [16] investigated experiences of belonging in both general and gay communities within Australia, with a focus on predictors of depression among self-identified gay men (n = 137). Their findings indicate that an increased sense of belonging is associated with decreased levels of depression reported by gay men [16].

### Mental health, minority stress, and sexual citizenship

Minority stress theory [17, 18] proposes that mental health disparities can be explained in large part by stressors induced by a hostile, stigmatizing, homophobic and transphobic culture, which often results in a lifetime of harassment, mistreatment, discrimination and victimization. 2SGBQ + men have been shown to be at an increased risk of psycho-social stress factors and negative mental health outcomes, including various forms of anxiety, depression, complex trauma, and suicidality, the suffering of which is exacerbated by experiences of social isolation, disconnectedness, and discrimination [17, 19]. “Stigma-based stress”, for sexual minorities, involves several facilitators that compound on the stress experience itself [17]. Social judgment, blame, prejudice, and oppressive economic and legal policies are some examples, where such compounding drivers also come to intersect with variables such as race and class, ultimately resulting in negative health and social outcomes [17]. Barriers to, or the explicit removal of, queer spaces and social connections that act as protective factors against stigma-based stress have therefore been shown to impact mental health [1].

At the same time, an established body of public health and social science research links social stigma with poor mental outcomes among sexual minority men, and considers how these linkages are mediated by place, and in particular place-based minority stress [20]. For example, research by McConnell et al. examined intersections of minority stress among sexual minority men (n = 33) in US. The authors found that minority stress is often intersected with specific identities and identity formation wherein “associated forms of minority stress were embodied in their physical appearance, situated in specific neighborhoods and contexts, and co-constructed through their interpersonal interactions with others” [18]. Research has also found that neighborhood characteristics significantly influence sexual minorities’ level of depression and overall mental health [21]. Stigma-related stress for 2SGBQ + men is explicitly felt within the context of health care access and patient provider interaction [5]. Furthermore, policy regimes, and the ways in which sexual minorities experience place relationships, ultimately contributes to overall mental health [20].

Current critical social theories surrounding biopolitics can supplement minority stress theory. Two concepts borrowed from biopolitics (biosociality and biocitizenship) complement each other and are conducive to studying the impacts of COVID-19 on 2SGBQ + men’s mental health. Biosociality is the idea that people with similar biological conditions actively form social and support networks [22]. It describes how different identities and social groups develop around new areas of knowledge [22]. Biocitizenship, on the other hand, goes beyond just belonging to a group. It also involves the ethical connection between that group and a state or institution. This connection includes certain rights, entitlements, and responsibilities that group members have towards each other [23]. Both biosociality and biocitizenship provide broader frameworks for understanding sexual citizenship. While sexual identity is a central aspect of sexual citizenship, unlike biological conditions or diseases, there are points where sexual citizenship and biosociality intersect. For example, during the AIDS crisis of the 1980 and 1990 s, queer men actively organized to improve access to safe treatment for HIV/AIDS. This shows how sexual citizenship and biosociality can come together [24]. To better capture the interplay of rights, responsibilities, sex, sexuality, health, and disease, the term “biosexual citizen” combines the concepts of sexual citizenship and biocitizenship [23]. It reflects the interconnectedness of these ideas and their impact on individuals’ lives. Minority stress is closely tied to how 2SGBQ + men can express and engage with their own identities as biosexual citizens. This becomes especially crucial when they are denied access to community connections, safety, and spaces associated with sexual citizenship. When 2SGBQ + men are unable to access safe spaces, they are also excluded from community participation, which can worsen existing mental health disparities.

2SGBQ + men face challenges in navigating the boundaries of sexual citizenship, moving between legality and illegality, societal silence and celebration. The COVID-19 pandemic has highlighted new borders, including access to safe queer spaces, while raising broader questions about mental health, community involvement, and minority status. Examining critical responses to the pandemic’s impact on 2SGBQ + men’s mental health within the context of biopolitics, we argue for understanding these responses within changing sexual politics of community belonging and social inclusion. Furthermore, the broader role of ‘place’ in 2SGBQ + men’s mental health remains under-theorized due to a lack of qualitative research in partnership with this community in the province of Manitoba, which includes engagement with critical social theory on space/place dynamics and associated connections with informal citizenship and identity. The forced closure of queer events, physical meeting spaces, and restrictive access to 2SGBQ + friendly health and social care facilities during the COVID-19 pandemic, combined with isolation from social support networks, may intensify the psycho-social and mental health needs of an already struggling population and location. Yet, very little research has focused on these impacts of COVID-19 on 2SGBQ + men within the province.

## Methodology

### Study design, recruitment, and eligibility

The data utilized in this paper comes from the qualitative phase of a sequential mixed-methods, community-based study focusing on the lived experiences of 2SGBQ + men in the province of Manitoba during the COVID-19 pandemic. This study was conducted under the guidance and consultation of a community advisory committee (CAC) that consisted of nine 2SGBQ + men from varied geographic communities, economic backgrounds, and educational experiences. The CAC provided integral support to the research team during the development and implementation of the study.

Participants were recruited from the cities of Winnipeg, Brandon, Steinbach, the Pas, and Thompson and were notified of both the quantitative and qualitative phases of the study using flyers posted at service providers, online through social media platforms (Facebook, Instagram), and word of mouth. Participants (n = 366) were first invited to take part in an online survey as part of the initial quantitative phase of the study. Through this method, participants were then free to indicate an interest in a subsequent qualitative interview. 2SGBQ + men who specified that they would like to participate in the qualitative phase of the study (n = 18) were then contacted by a member of the research team. All other interviewees (n = 2; those who did not take part in the online survey) contacted a member of the research team directly to set up an interview time.

Participants were screened for eligibility prior to the interview. Eligibility criteria used to screen potential participants included the following: [1] identify as a man (cisgender or transgender), [2] report any sex with another man in the previous 12 months and/or identify as two- spirit, gay, bisexual or queer, [3] be 18 years of age or older and [4] live or work in Manitoba. This study was particularly concerned with showcasing diverse voices from around the province, which included members from key populations such as Two-Spirit and Indigenous GBQ + men, 2SGBQ + men living in rural settings, 2SGBQ + men living with HIV/AIDS, and 2SGBQ + men with substance dependence and substance use.

### Data collection

Semi-structured interviews were conducted between May – July, 2020 with 20 2SGBQ + men via online video conferencing platform (Zoom) and via telephone. Informed consent was obtained prior to each interview. Participants were provided with monetary compensation equaling $45 (CAD) for their involvement. The semi-structured interviews included the following questions: “How would you generally describe your mental health? Did COVID-19 affect your mental health? Since the COVID-19 pandemic began, how has your mental health changed? What specific changes have you noticed, and how are you taking care of yourself? How are you managing self-isolation during COVID-19? Where do you usually seek mental health services?”

### Data analysis

Audio recordings of each interview were then transcribed and analyzed using the data analysis platform MAXQDA [25]. A master code list was initially generated from an interview guide, and then later grew to include inductive codes that emerged from the coding process. An initial pass of coding the first 3 transcripts was conducted by all three team members to assess for inter-rater reliability, define and clarify code definitions, and establish new inductive codes and code rules. Upon completion of the initial pass, team members divided up the coding work to complete the analysis process. Our analytical procedure involved careful line-by-line reading and analysis of the transcripts, and iterative analysis to understand 2SGBQ + men’s meanings of their experiences, lived worlds, and significant themes related to mental health, and community belonging. Coding and analysis were checked for consistency in several research team meetings and discussions. Once all transcripts were coded, members of the research team used thematic analysis to create explanatory models that subsequently gave rise to the conclusions presented below. Specifically, data analysis involved delineating units of meaning from the data, clustering units of meaning to form thematic statements, and extracting themes. Member checking occurred with CAC members (two meetings) and service providers (one meeting) to explore and improve the credibility of study data, which involved sharing emerging themes and participants’ quotes with CAC members. Member checking allowed CAC members to inform the themes emerging from the interviews. Reflexivity was maintained through on-going documentation in memo trail, core research team meetings, and comparing this reflection against the data in the analysis process.

## Results

### Sample characteristics

The sample included 20 2SGBQ + men in Manitoba, with an average age of 35. A significant proportion (n = 13; 65%) identified as White. In terms of gender identity, the majority of the 2SGBQ + respondents identified as cisgender men, with three individuals identifying as trans and two individuals identifying as Two-Spirit.

### Results overview

The results highlight the relationship between social isolation and mental health outcomes, the loss of physical and social spaces and places in Manitoba, and the social functional role of queer public spaces for 2SGBQ + men’s communities. Our results describe the exacerbation of pre-existing inequities and gentrification of queer spaces. Four key themes are presented below.

### COVID-19 restrictions and social isolation negatively impacted 2SGBQ + men’s mental health

Social isolation was cited as one of the most common causes of mental distress among participants, with three respondents explaining:

*It’s been horrible. I’m a very social person, we both [referring to partner] are and the inability to socialize has been very hard on us, ridiculously hard. I’m back on antidepressants after having been off them since 2012 because I found I was having a little bit of trouble coping. There are some elements. My depression usually doesn’t have an element of anxiety, but my anxiety has been really high about it too. It’s not so much worrying about contracting COVID or knowing somebody contracting it. It’s more about the social isolation. It’s just been horrible for us both.* 47 years old/Cis/Gay/Caucasian

*Right. I’m somebody who, I don’t cry a lot but last year in December, when things were really bad, I was crying more days than I wasn’t just because I was just feeling so alone all the time. So, it was very difficult, probably the hardest time in my life I would say. 20 years old/Gay*

Participants described the sudden shifts and challenges they faced as the world rapidly changed due to the pandemic. As the following quote exemplifies, for some individuals, the stress of adapting to these changes resulted in a relapse:

*It took a really tough turn. I was actually … since 2015, I have been dealing with a crystal meth addiction. I was six months, seven months sober, and then the pandemic hit, and then that really changed everything because all my meetings, my AA meetings and stuff, were gone right away. They were online, but it was way different. I definitely relapsed then, and that was right at the start of the pandemic. 28 years old/ Agender/Gay/Caucasian*

The intensive loss of both formal and informal support groups, as well as the loss of access to social gatherings and friend groups were often discussed within the context of abrupt changes to everyday living. Such drastic changes, whether to social life, access to health and social services, or to work and employment, were expressed as particularly difficult given the dynamic nature of restrictions and gathering protocols. Participants often sought out several coping strategies to mitigate the mental distress that came with such abrupt shifts in their social worlds. For many participants, coping often took the form of resistance to gathering restrictions altogether:

*The hardest part for us was being isolated but we only did that for about three months. And we would see a small group of friends, we have a core group of about 12 friends that we would, I guess you could say, illegally see. Probably we phased ourselves into it by not seeing them much, like maybe once a month we would see somebody. But even now, with the restrictions we have in Manitoba you can’t go anywhere or go to somebody’s house, we still do see people once a week. There is a great deal of benefit to us seeing people, so we had to kind of weigh it out. So, we did see quite a few, we did see people often but not a large group of people. 47 years old/Cis/Gay/Caucasian*

Such balancing “of the benefit” was often a point of reflection for those participants who chose to obfuscate restriction policies to reduce mental distress, depression, or anxiety. Conversely, involvement in prohibited gatherings also created anxiety and apprehension as much as it served to alleviate long standing forms of isolation. While many participants openly discussed their involvement in rejecting social distancing to maintain social bonds, some participants also described how acceptance of social distancing measures served to create a sense of community, understanding, and empathy around shared isolation, which ultimately helped to alleviate feelings of distress:

*No, no, it wasn’t always bad. I would say that the bad times got worse, but the fear of missing out has been relieved because no one could go anywhere or do anything for a while. I go for walks every morning, and when I see people together and they are with a lot of friends, it makes me feel bad because I don’t have that. But when I would go for walks during the pandemic and people were just walking by themselves, or most people stayed home, I didn’t have that loss of missing out. That has eased a little bit, as well as the isolation, because anyone I would talk to says, well, they are just binging Netflix. I said, yes, that’s what I’m doing, too. I don’t feel as odd by staying home all the time. 52 years old/Gay/Cis*

### 2SGBQ + community experienced loss of safe spaces due to the COVID-19 pandemic

Many respondents espoused the view that safe queer spaces were disappearing or transforming, thus further perpetuating social exclusion of 2SGBQ + men in Manitoba, particularly the marginalized members of the community. The quotes in this section illustrate the situation regarding participants’ experiences and perceptions of the disappearance of the gay community in Winnipeg due to the COVID-19 pandemic. The following quote exemplifies a common response from study participants, highlighting the connection between social exclusion and the disappearance of queer social spaces:

*I think we’re being pushed more into the margins because even our social spaces are disappearing. 28 years old/ Agender/Gay/Caucasian*

Some respondents commented on the absence of socializing with other queer people, highlighting the importance of community on queer identity, and discursively making links between reduced access to queer safe spaces and isolation from community and friends:

*When you’re queer and you haven’t seen your community or been around your community in such a long time, I feel like that’s the thing I’m missing the most is just being around other queer people….There’s a community in Winnipeg that’s a pretty solid queer community, like a lot of sort of social activism and there’s a really cool drag community… I realize that that was a pretty important part of my identity as a queer person was just being able to be part of those events and support marginalized people in our community and stuff. And now it’s like that just hasn’t happened for so long and it’s a little bit lonely I guess….I know that for my very close friends, they’ve expressed the same thing, just feeling a little alienated or lonely or whatever since the pandemic started, just missing other queer people…it’s like when you’re queer, you make your own family sometimes. Not having access to that in spaces that are safe like gay bars, it’s really challenging for people to not have that. When it’s absent, it’s something you miss for sure. 42 years old/Cis/Gay/Caucasian*

Other respondents also shared how the loss of safe spaces (spaces free from homophobia, transphobia, and other oppressions) during the stay-at-home restrictions made them confined in hostile home environments with unsupportive, homophobic family members:

*As a queer community, as a marginalized community, strength in numbers is super, super important for us, and we don’t have that right now. We’re all just in our little silos, and then we have people like me, and I know I’m not the only one who is living with family members who are deeply homophobic, and so it just exacerbates everything else that we were already feeling so we don’t have that community. The medical system is taxed, and exhausted, and is homophobic to begin with. These resources in the community that were there are far less available… The groups were cancelled, and then they were moved online, but it’s just not the same thing. 32 years old/Cis/Gay/Caucasian*

The findings also reveal how the profound effects of the gradual disappearance of safe spaces for some marginalized members of the 2SGBQ + men’s community – homeless people:

*Let’s say 2019, it was packed, we were doing everything. Pride was packed…And then, COVID hit, and for a while…we were still meeting each other because we didn’t really know what was going on. But then…once the bar closed, we had no forum to meet up or hang out or do shows. Thank God, I also am involved with the [agency], and they are amazing. Once COVID hit, they kind of ramped up and took charge of it. It’s okay if you need something, just drop in, 15 minutes, that’s it, and you can go, and there was a rotating door. The space that we had there, which is like that, every time I came, it was always very inclusive. We deal with a population that is … the homeless population. Some people that do show up are very, very, let’s say, not queer-friendly, or they don’t know what is going on or who is there. Slowly, I started noticing that it wasn’t a gay drop-in anymore. There were three queer people, and then the rest would be just drop-in drop-ins….Once you start putting our safe space that we created in jeopardy by saying ‘faggot’, ‘these fucking faggots’, blah, blah, blah, queer this … Slowly, I started feeling very unsafe there. 37/Cis/Gay/Salvadorean*

Some respondents also commented on how the disappearance of social spaces exacerbated the social exclusion of people who were already disenfranchised from within mainstream gay/queer community in Winnipeg, such as Indigenous people:

*Like holy shit, queer people are my family, but I can’t see them anymore because the spaces are closed. I also think spaces might even become less accessible to people, like gay bars. We only have one in Winnipeg … and it’s inaccessible to people who don’t feel like they’re part of the queer community, like Indigenous people. 42 years old/Cis/Gay/Caucasian*

### The COVID-19 pandemic revealed the social function of queer public spaces

Many participants often commented on the social function of public spaces for 2SGBQ + men, highlighting their role as places of refuge and self-expression, which were now being impacted by the COVID-19 pandemic:

*Lots of them [2SGBQ + men] were finding their relief going to the bathhouse or something like that because this is the only space where they can actually express themselves at. I think they are serving a certain function within the community…. people are still not quite there yet on going out as they used to go before COVID-19. 55 year old, Jewish, cis gay man*

Many expressed concerns over disappearance of (already segregated and gentrified) physical meeting places for 2SGBQ + men (served a function of meeting other like-minded people), and commented that Pride events were taking a more subdued form during the pandemic:

*The queer community in Winnipeg is kind of weird. When you go into a lot of other cities, and there’s definitely a core, especially in the big cities, like, Montreal, Toronto, and Vancouver. There’s a very clear gay village. Winnipeg doesn’t have that. It’s always been just kind of scattered…I’m concerned that we’re going to lose a lot of those meeting places…And then all of the restrictions are going to end, but we’re still going to be restricted because those places are going to be gone, and that’s sad. If you look at what they’ve tried to do with Pride this past year, Pride Winnipeg, hats off to them, they tried, but it paled in comparison to the normal thing and sure, it would. Yeah, it’s going to be a different world in all sorts of different respects, but especially with this community…For me, having those community spaces was super helpful to have other like-minded people... 32 Years Old/Cis/Gay/Caucasian*

Some respondents expressed optimism that while community spaces may be transformed due to the pandemic, they will still remain available after the pandemic is over (once again, highlighting the importance institutional role that these spaces create for community members):

*I think that the social spaces that we have are going to remain the same. Like the bars that we have, the bath house, people seem to want to keep these. I have seen on social media people supporting them with fund raising money to pay their rent and stuff. So, I think that gay people want to keep the spaces that they have… COVID is going to … transform some things, like if they may locate to another location but I think all the services that have been available will remain after COVID. 20 years old/Gay*

### COVID-19 pandemic exacerbated inequities and gentrification of the queer scene

Many respondents commented that the discrimination and stigmatization of queer people existed before the pandemic, and how the COVID-19 pandemic exacerbated many pre-existing inequities for community:

*I want to put an emphasis on accentuated systemic discrimination, stigmatization, or whatever you want to call it that existed before. All of that inequity that queer people face has been an issue that’s as old as time, and yet, I think the pandemic just exacerbated those things, made them worse. 32 years old/Cis/Gay/Caucasian*

Respondents also talked about how gentrification of the gay scene started before the COVID-19 pandemic (including the proliferation of online hook-up devices, or the disappearance of gay men at gay bars) and how the pandemic exacerbated and facilitated these processes:

*I think prior to the pandemic, there was a period of gentrification of our scene. I wasn’t quite comfortable with the bar scene prior to the pandemic, and then, of course, the bars closed. I think we only had one that I would be comfortable going to, but it closed for a lot of the pandemic. I think the apps… people are using apps to meet people for hook-ups, but that started, again, prior to the pandemic. I think the pandemic just sort of cemented that this is the new way that people meet. 52 years old/Gay/Cis*

Others also commented on how gentrification and segregation of the physical queer community in Manitoba was taking place, and how the physical venues in the gay community or Pride events were already being taken over by non-queer people before the pandemic started:

*I guess my hope is that there would be, maybe, a bar spring up or more groups, more activities. There might be such a relief that the pandemic is over that there will be more opportunities. My fear is that it will revert back to pre-pandemic times, where everything was done by apps and our community was gentrified. It was taken over by straights, really, or bars became … gay bars used to be such a safe space for the gay community, and then they turned into gay-themed bars for straight people. Part of my sadness is that there are limited gay community spaces in Winnipeg. Even the Rainbow Resource Centre, I find that … I identify as a gay man. I was born a gay man in my experience. But see, I think there is only one group every second Sunday for gay men. In other cities, there would be more. They would have something daily, several options daily, and unfortunately, in Winnipeg, I don’t have that option….I also feel, with Pride, at one time, it meant something. It was … when I came out, we didn’t want to be fired from our jobs or denied an apartment, and I had experienced both. We didn’t want to be bashed in the streets, and I had experienced bashings. Now, if I tried going to a Pride … it was a lot of straight people. 52 years old/Gay/Cis*

## Discussion

Findings from this study underscore existing research that highlights the deleterious impacts of the COVID-19 pandemic on the mental health of 2SGBQ + men in Manitoba. Results also highlight the importance of access to safe spaces for 2SGBQ + men while contributing to the body of research related to the loss of social connections due to the COVID-19 pandemic. First, safe spaces were by and large lost by many of the participants in this study with pronounced effects to their social and mental health. For example, participants spoke of losing opportunities to engage in activism while also being required to spend time in unsafe situations with families, roommates, etc., who were not supportive of their identities and/or sexualities. In our view, the COVID-19 pandemic has increased stigma-based stress for participants as outlined by Banajeree & Nair [17], and Petruzella et al. [18]. Moreover, the health-promoting aspects of queer socialization were also hampered or at least interrupted through the shift to virtual meeting and group opportunities. While this may have led to a reduction in health risk outcomes, as outlined by Buttram and Kurtz [15], we are cautious to evaluate the value or cost of the sacrifice made by the participants in this study to follow public health guidelines to prevent the transmission of COVID-19. This leads to one area that needs to be explored further, and that is to fully evaluate the impact of reduced social connections during COVID-19 on both mental health and sexual health outcomes post widespread social lockdowns.

With regards to place-based minority stress, our findings are also consistent with work by McConnell et al. [20], which describes certain forms of minority stress as embodied, situated, and co-constructed. For 2SGBQ + men living and working within Manitoba, the physical and symbolic removal of safe spaces creates a form of situated stress that is dependent on lack of access to community, thereby creating exposure to stigma, discrimination, and violence within other settings. This form of minority stress demonstrates a “setting-level” [20] form of intersectionality and thus has considerable effects on both identity formation and maintenance as well as the formation of social connections. Experiences of minority stress were also co-constructed in relation to participants’ interpersonal interactions, or lack of thereof, with peers, family members, community leaders, and health care providers. Discrimination from family members and healthcare providers were mentioned as particularly difficult, with this form of co-constructed stress intersecting with the above given the lack of access to spaces that provided a haven from such interactions.

In general, our study brings forth significant contributions to the existing literature on the impact of the COVID-19 pandemic on the mental health of 2SGBQ + men. Firstly, our findings highlight the profound intensification of stigma-based stress experienced by the participants because of the pandemic. Additionally, our study uncovers the detrimental effects of pandemic restrictions on the vital health-promoting aspects of queer socialization. Furthermore, our research contributes to the ongoing discourse on place-based minority stress by elucidating a distinct form of situated stress resulting from the closure of safe spaces for 2SGBQ + men in Manitoba.

Our results also build on current critical social theory centered around the concepts of biocitizenship, biosociality, and sexual citizenship [26]. The COVID-19 pandemic acted as a primary driver in shaping spaces of sexual citizenship for 2SGBQ + men in Manitoba. Our findings revealed the deleterious effects of having little or no access to public spaces of sexual citizenship, and the consequential effects of these pressures on 2SGBQ + men in Manitoba during the COVID-19 pandemic.

The COVID-19 pandemic, in re-organizing access to (and definitions of) spaces of sexual citizenship, also profoundly changed the ways that 2SGBQ + men came to identify, or reaffirm their identities as, ‘biosexual citizens’ within peer-led, activist, and other social communities. As physical spaces of meeting and organizing transitioned online, or disappeared altogether, access to formal and informal spaces came to dictate new modes, experiences, and expectations on the responsibilities and rights of membership within specific biosexual groups. In the context of COVID-19, new emergent biosocialities became actualized in the context of pandemic experiences for 2SGBQ + men, which includes both shared illness experiences and the collectively felt effects of exclusion from private and public spaces of sexual citizenship. As in the case of Hakim et al [23], our results suggest that 2SGBQ + men within Manitoba came to develop a number of “creative strategies that combined their desired forms of sex and intimacy with their efforts in enacting biosexual citizenship so as to avoid further diminishing their quality of life, already diminished by living through the difficulties of a global pandemic” [23, 298]. Queer men within Manitoba accepted or rejected pandemic restrictions to secure access to informal social groups that provided a sense of support, acceptance, and safety, especially in cases wherein private home life proved to be unsafe. Such adaptations can further be framed within what Bradley [22] refers to as ‘biosolidarity’, where “through the meaningful connections and care networks found in the biosocial group”, resilience and resourcefulness in the face of adversity is manifested [22].

2SGBQ + community members who participated in our study also shared concerns related to the shifting landscape of social spaces in Manitoba. As mentioned above, Winnipeg was regarded as a social hub for queer communities in throughout Manitoba and in neighboring provinces. However, over time, gentrification and the shift to online socialization (as is the case with many queer spaces globally) have deteriorated the need for such physical locations leading to a reduction of meaningful place-making for this community. It is also important to note that these impacts are not felt unilaterally by members of the 2SGBQ + community; those who are considered the most marginalized are often in positions to feel these losses more severely. With this in mind, our results support the work of Ristock [13] and McLeod [5] in articulating the continued erasure of physical spaces for the creation, maintenance, and celebration of queer identities.

### Limitations

Because of the extremely diverse and non-representative sample consisting of twenty 2SGBQ + men, it would be a mistake to generalize about any group or make any wide-reaching cultural comparisons between specific identities. For example, Indigenous Two-Spirit people within the queer community in Manitoba also face added oppression and experiences of discrimination due to the continuation of colonial control and governance. The diversity of 2SGBQ + populations require tailored strategies for these groups. We also acknowledge that the fact that most participants were over the age of 35 may impact the generalizability of our findings and their broader applicability. In addition, confirmation bias may have influenced data collection, analysis, and interpretation. Steps were taken to address confirmation bias, including engaging in the practice of reflexive thematic analysis, member checking the findings with community members, and involving multiple interviewers as part of our data collection methods.

### Recommendations

Health professionals who work with 2SGBQ + men should pay attention to the negative impacts of social isolation on mental health to better engage these men in health promotion activities or when designing services. Health intervention efforts may seek to focus on increasing culturally sensitive and affirming mental health supports, programs, and services. Service providers, including doctors, nurses, social workers, and other professionals involved in the provision of mental health services and care, need to be informed about the unique mental health needs of 2SGBQ + men in Manitoba and identify coping strategies and resources to deal with negative mental health outcomes accrued from the COVID-19 pandemic. From a holistic perspective, interventions to improve access to economic and social resources designed with the unique needs of 2SGBQ + men may also be required to improve this communities’ mental health. Furthermore, to mitigate the impacts of COVID-19 on depression, anxiety, and social isolation on 2SGBQ + men, service providers also need additional training to meaningfully address the discrimination, stigma, oppressions, and social exclusion that perpetuate minority stressors for this group. From a systemic perspective, primary and secondary care (psychologists, counselling, social workers, specialists) need to adequately meet the needs of 2SGBQ + men by seeking out 2SGBQ + specific training to learn how to remove and reduce barriers in mental health access for this population.

Our research also highlighted some potential links between 2SGBQ + men’s mental health and their social and physical environments. While COVID-19 restrictions have eased in Manitoba, it is still essential that 2SGBQ + men are provided with spaces, organizations, events, support services and opportunities for socializing aimed at counteracting the negative impacts on mental health that they accrued during the pandemic. It is important that 2SGBQ + men are provided with opportunities to connect and find intimacy with members of their communities. This includes supporting safe community spaces/places, events, and community organizations that cater to 2SGBQ + men’s communities financially, socially, and politically. Queer spaces (both social and physical) enable social connections within 2SGBQ + men’s communities and are important for 2SGBQ + men’s mental health because they serve as safe havens within mainstream heteronormative Canadian society. Queer safe spaces are also crucial for providing a sense of social inclusion, identity, and community belonging as well as being key platforms for delivering mental health promotion and services. Continued financial investment in such spaces which allow 2SGBQ + men to safely express themselves and re-establish their connections and social networks is recommended.

Finally, these findings also highlight the need to address stigma, promote social equity, and eliminate discrimination of 2SGBQ + men that existed prior to the COVID-19 pandemic. Structural changes, community interventions, and public-awareness campaigns are essential for reducing disparities and improving mental health outcomes for 2SGBQ + men.

## Conclusion

During the COVID-19 pandemic in Manitoba, 2SGBQ + men experienced a marked loss of social connections, community spaces, and social networks which are specific to their socio-sexual identities. This, in response, has intensified pre-existing mental health disparities. The findings highlighted how COVID-19 restrictions have reinforced the value of close personal communities, families of choice, and social networks among 2SGBQ + men in Manitoba, Canada. The research points to important role of safe community spaces, events, and community organizations on 2SGBQ + men’s mental health. Utilizing theoretical constructs of minority stress and biopolitics, the findings from this study point out the political role and utility of safe queer community spaces, places, organizations, and events for 2SGBQ + men’s mental health.

## Data Availability

The datasets used and analysed during the current study available from the corresponding author on reasonable request.
